# Fast Handover Algorithm Based on Location and Weight in 5G-R Wireless Communications for High-Speed Railways

**DOI:** 10.3390/s21093100

**Published:** 2021-04-29

**Authors:** Baofeng Duan, Cuiran Li, Jianli Xie, Wei Wu, Dongmei Zhou

**Affiliations:** School of Electronic and Information Engineering, Lanzhou Jiaotong University, Lanzhou 730070, China; 0117046@stu.lzjtu.edu.cn (B.D.); xiejl@mail.lzjtu.cn (J.X.); wuwei@mail.lzjtu.cn (W.W.); zhoudongmeitd@mail.lzjtu.cn (D.Z.)

**Keywords:** rail transit communication, smart railways, handover chain, delay of the handover, success rate of the handover

## Abstract

With the booming development of high-speed railways (HSRs), key technologies of wireless communications need to be constantly innovated. In particular, the frontier issue of low delay of the handover for the fifth generation (5G) in fast-moving scenarios has attracted attention from both industry and academia. Based on an analysis of a large number of measured data and the location of the user equipment (UE), a fast handover algorithm is proposed to solve the problem of long delay for a train moving at high speed in a 5G-railway (5G-R). By calculating the speed of a train and its direction of movement, a reasonable handover mode is selected and the handover chain of neighboring cells is identified. The location of the train can be calculated to determine whether UE enters the defined identification zone of pre-handover. Depending on the values collected in the measurement report, the command of the handover is triggered when the weight of the target cell is greater than that of the source cell. Our experimental results show that the delay of the fast handover algorithm is reduced by 2.03%, and the success rate of the handover is increased by 0.42%. Research directions for smart railways are discussed based on these findings.

## 1. Introduction

China has developed high-speed railways (HSRs) with 38,000 km in total length, accounting for over two-thirds of the world’s total [1]. One railway was built with a speed of 350 km/h to enable athletes, reporters, audiences, and service staff to shuttle efficiently between Beijing and Zhangjiakou for the Beijing 2022 Winter Olympics. In the meantime, HSRs are under construction all over the world. In recent years, wireless communications applying the long-term evolution for railway (LTE-R) have been under consideration [2,3,4,5]. However, its maximum bandwidth is just 20 MHz, which can only maintain tens of Mbit/s data rate and cannot meet the requirements of Gb/s-level applications [6]. As mobile networks of the fifth generation (5G) are being deployed rapidly, higher data rates, lower communication delays, and more device connections are expected to be achieved by 5G for railway (5G-R). However, a speed above 200 km/h of a high-speed train (HST) requires more frequent handovers and a shorter time to pass through the overlapping coverage area of two neighboring cells [7]. Moreover, it may lead to serious deterioration of wireless communication indicators, such as the delay of handover and the success rate of the handover, and this is similar to LTE-R [8,9]. Wireless network optimization can effectively eliminate the interference of communications along the rail, avoid the existence of no-signal areas, and improve a user’s perception [10,11]. Due to the high speed of user equipment (UE) on a train, wireless communications of 5G-R face great challenges [12,13]. Therefore, the development of fast handover algorithms for HSRs is essential in support of speeds of 200 km/h and for use of 5G-R.

In order to reduce the delay of the handover of wireless communications, several different approaches have been explored. A simple but effective algorithm of distributed load balancing is presented to reduce the service interruption ratio due to frequent handovers in high-speed scenarios [14]. Adjusting power in the time domain relieves the “uncertainty” of the received strength of the signal and requires less energy transmission in the procedure of handovers [15,16]. The latest research on C/U-plane staggered handover suggests a methodology to achieve soft and fast handovers [17]. With the large bandwidth of free-space optical communication, the loss rate of data can be decreased and the handover performance of the communication for HST is improved by changing hardware structures [18]. The optimal distance from the rail of a train to a ground base station and the distance between base stations are found to provide seamless connectivity and handover while minimizing the number of stations along the rail [19]. The optimization strategy of the handover based on the upper bound algorithm [20,21], which finds the station with the largest reward by sampling from the reward set of stations, can effectively improve the performance of the handover. However, when UE enters a different scenario, the algorithm of upper bound needs to be drawn from a random initial point again. It is also required to learn from a random initial point for a newly connected UE. Because the performance of the initial random point is poor, it may cause the communication quality to be lower than what the system requires for a long time. This is unacceptable in practical applications. Due to the penetration loss of HST [22,23], complex coverage scenarios, frequent handovers, and doppler shifts in HSRs [24,25], the architecture of a network has a great impact on the performances of wireless communications. However, existing handover algorithms are difficult to deal with in highly dynamic, complex scenarios, and special channel environments [26,27,28]. This has become a limitation of mobile communication technology for HSRs. While previous studies made connections between the handover and system performances, they improve only one of performances while worsening another. Some studies suggest increasing the cost of construction for wireless communications to reduce spacing of base stations in order to obtain favorable indicators of communications; few studies involve the optimization of the overall performances with the fast handover algorithm as the pointcut under the condition of the existing size of base stations.

**Figure 1:**
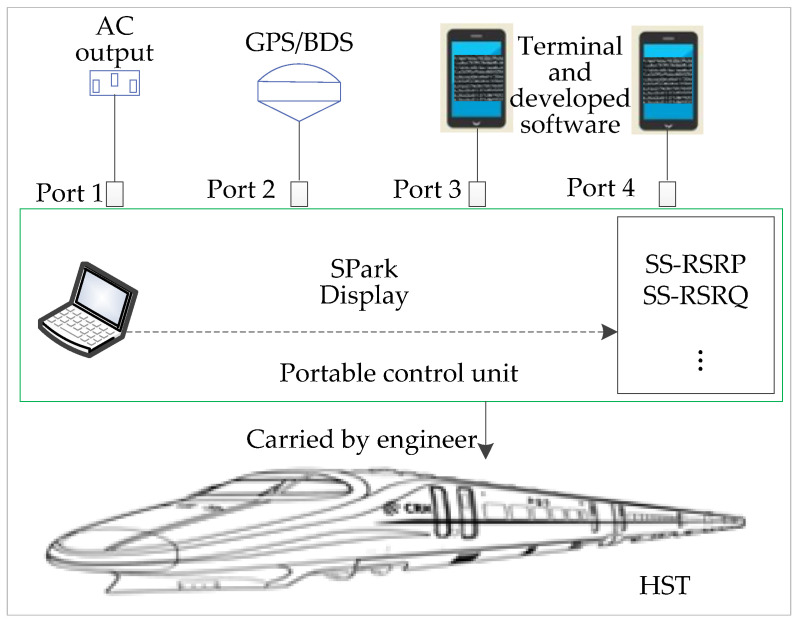
Principle of collecting massive amounts of measured data.

This paper takes the wireless communications of 5G for HSRs as an application scenario to analyze system architecture, planning of the handover zone, synchronization signal reference signal receiving power (SS-RSRP), and quality (SS-RSRQ) measurement of the serving cell (SCell) and the neighboring cell (NCell). The collection of the measured data with location information can be acquired by the portable control unit (PCU). It is composed of five ports to connect external devices, as shown in Figure 1. An alternating current (220 V) can be obtained from the train or from a portable power source. The terminals HUAWEI P40 Pro, installed with developed software to measure the wireless communications, are connected to port 3 and port 4. The software SPark can be used to display some of the test results in time, including the path loss, SS-RSRP, SS-RSRQ. A fast handover algorithm in HSRs is realized through the process of the speed decision, the location recognition, and the weight calculation. The results of this paper have important implications for designing wireless networks, constructing base stations, and improving performances of communications for 5G-R. The rest of the article is organized as follows: In Section 2, the architecture of 5G-R in HSRs is presented through the measured data, with the factors for handling the handover zone analyzed. Section 3 develops the fast handover algorithm in detail. The experimental results are presented to verify the effectiveness of the proposed algorithm in Section 4. Finally, Section 5 concludes with some concluding remarks and discussions.

## 2. G-R for HSRs

### 2.1. Architecture of Networks

**Figure 2:**
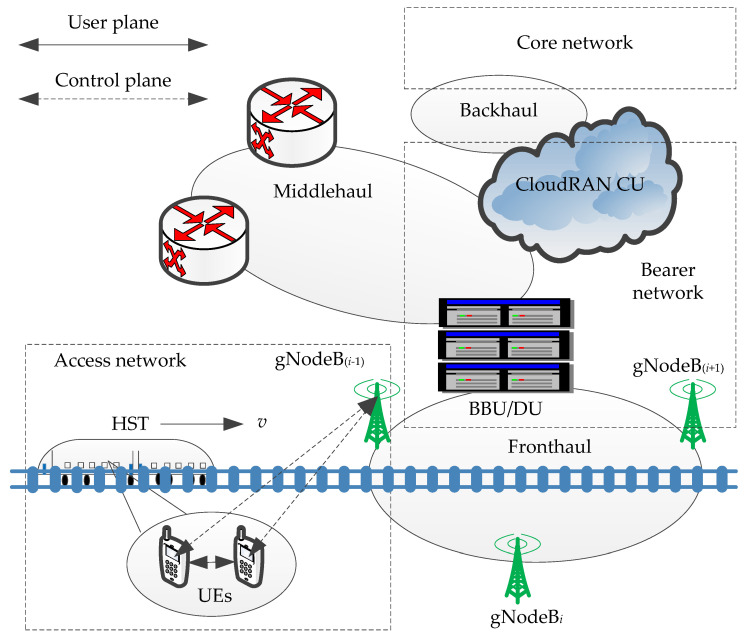
Architecture of networks in 5G for railway (5G-R) for high-speed railways (HSRs).

The architecture of networks in the 5G-R for HSRs mainly involves three parts: access network, bearer network, and core network, as shown in Figure 2. The smooth and stable mission-critical communication services are supported, such as signaling, onboard video surveillance, driver look-ahead as well as supporting passenger high-data-rate connectivity [29]. The building baseband unit (BBU) or the distributed unit (DU) offers large-scale cooperative processing and is able to achieve lots of real-time connectivity. The fronthaul network consists of gNodeBs and BBU/DU through a high-capacity optical fiber. The network decouples completely the control plane and the user plane, then reconstructs flexibly the system control. CloudRAN is introduced to address the requirements for the reconfiguration of the access network. If there is a mobile edge computing server, it is usually deployed in the same set of servers as the centralized unit (CU). In terms of the physical level, the bearer network is divided into fronthaul, middlehaul, and backhaul. The concept of the logic of middlehaul for the bearer network is proposed. The cellular structure is changed to the chain structure in a private communication system for HSRs because each gNodeB contains two sectors, while a public communication system typically consists of three sectors. The HST runs at an average speed of *v* and passes gNodeB_(*i*−1)_ (*i* = 1, 2, …, *N*), gNodeB*_i_*, and gNodeB_(*i*+1)_ in turn.

**Figure 3:**
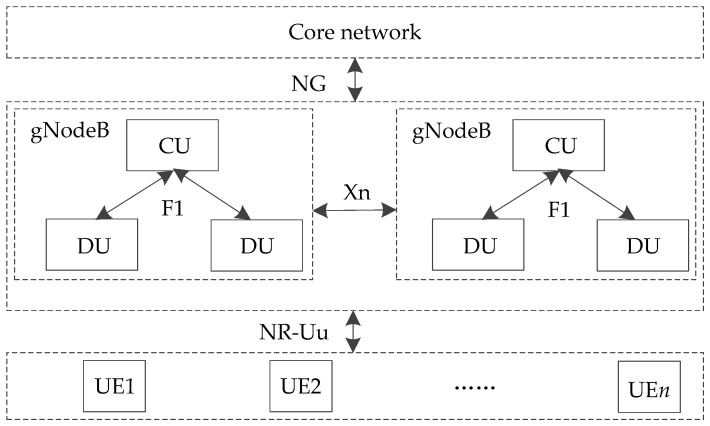
Interfaces between elements in 5G-R for HSRs.

As shown in Figure 3, there are corresponding interfaces to connect each element in 5G-R for HSRs. NG is the interface between the access network and the core network. F1 is the interface between the functional entities of CU and DU, which supports the signaling exchange between endpoints and the data transmission of each endpoint. The air interface of 5G-R is Uu, which is the port between UE and gNodeB. In order to distinguish it from LTE-R, it is defined as NR-Uu.

### 2.2. Planning of the Handover Zone

**Figure 4:**
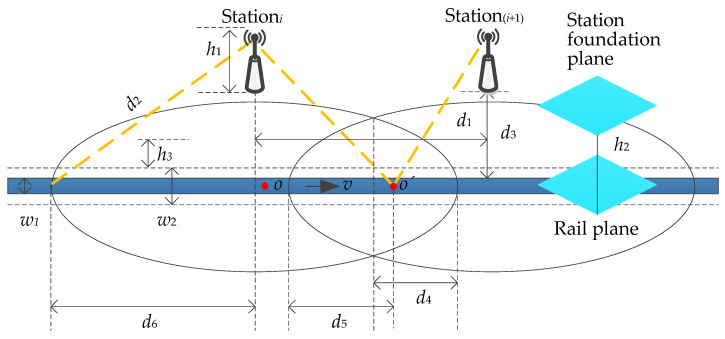
Model of the handover zone.

The model of the handover zone is established as shown in Figure 4. The width of the rail is *w*_1_ with the value of 1435 m (the international standard gauge), and the width of the HST is *w*_2_ with the value of 3328 m. The overlapping area of station*_i_*, station_(*i*+1)_, and the range within *w*_2_/2 on both sides of the central axis of the rail is the handover zone of the wireless network. The effective height of the station*_i_* is

(1) $$h=h_{1}+h_{2}-h_{3}$$

where, *h*_1_, *h*_2_, and *h*_3_ respectively denote the height of the station, the vertical distance between the station foundation plane and the rail plane, and the vertical distance between the geometric center of the train’s window and the rail plane. The station spacing *d*_1_ is the distance between station*_i_* and station_(*i*+1)_ as indicated in Equation (2):

(2) $$d_{1}=2\times (\sqrt{d_{2}^{2}-h^{2}-d_{3}^{2}}-d_{4})$$

where *d*_2_, *d*_3_ and *d*_4_, severally, represent the coverage radius of the station, the horizontal distance between gNodeB and rail, and the length of unidirectional overlapped coverage area. Suppose the point *o* is the location of the UE at any time and the point *o*’ is the handover location of two stations, then the distance that the HST travels with the handover hysteresis is

(3) $$d=d_{5}-d_{4}$$

Assuming the process of the handover takes Δ*t*, the planning distance of the handover zone is shown as Equation (4). As the moving speed of the train is known and the coverage capacity of cells is limited, the handover zone can be reduced by decreasing the delay of the handover, that is, the spacing between gNodeBs can be increased and the construction cost of 5G-R can be reduced. With the continuous raising of the speed of HST, the reduction of the delay of the handover can ensure that UE can complete the whole handover process within the given handover zone.

(4) $$D_{\mathrm{han}}=2\times d_{4}>2\times (v\times \Delta t)$$

**Figure 5:**
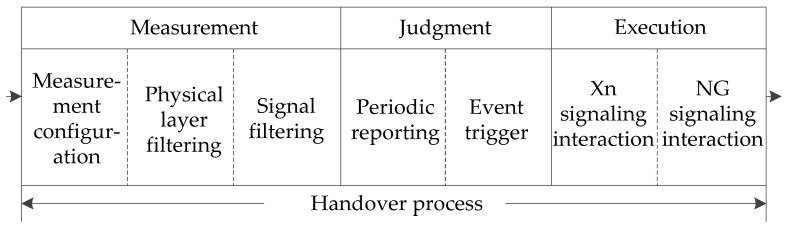
Diagram of the main process of the handover.

As shown in Figure 5, the process of handover consists of three phases: measurement, judgment, and execution. The measurement configuration message is the radio resource control (RRC) information sent by gNodeB to UE, including objects, identities, and types. Physical layer filtering and RRC layer filtering can reduce the jitter amplitude of measured values. The measurement is reported according to the interval cycle of measurement configuration parameters. When the Xn/NG signaling interaction is triggered when the rules of the handover are met, the Scell’s resources occupied by UE are released after the handover is completed.

### 2.3. Handover Measurement of SCell and NCell

#### 2.3.1. SS-RSRP

**Figure 6:**
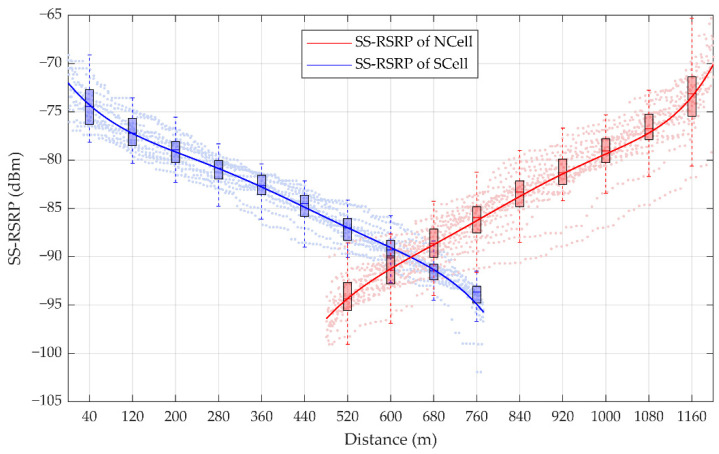
Statistics of the synchronization signal reference signal receiving power (SS-RSRP) measurement. The box plots represent the statistic of SS-RSRP in each 80 m: For a certain box, the bottom is the lower quartile Q1, the top is the upper quartile Q3, the horizontal line in the middle of the box is the median Q2; the top of the whisker is O3 + 1.5IQR, and the bottom is Q1 − 1.5IQR, where IQR is the interquartile range IQR = Q3 − Q1.

The command of the handover is triggered when the measured value of NCell is higher than the preset threshold of the measured value of SCell and this state lasts for a certain time. Two gNodeBs with a spacing of 1.2 km were selected in the section of an HSR from Lanzhou station to Jiayuguan Station in China. Based on the actual measurement data of a handover period, 1610 sampling points of the SS-RSRP of SCell and 1500 sampling points of the SS-RSRP of NCell are shown in Figure 6. According to the fitting curve, it is found that the measured values of SS-RSRP of SCell decrease gradually, while that of NCell increase gradually.

#### 2.3.2. SS-RSRQ

**Figure 7:**
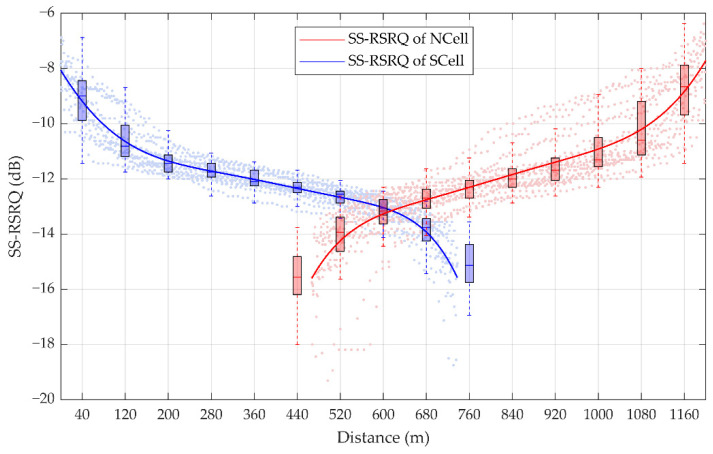
Statistics of the synchronization signal reference signal receiving quality (SS-RSRQ) measurement. The box plots denote the statistic of SS-RSRQ in each 80 m. For a certain box, the bottom is the lower quartile Q1, the top is the upper quartile Q3, the horizontal line in the middle of the box is the median Q2; the top of the whisker is O3 + 1.5IQR, and the bottom is Q1 − 1.5IQR, where IQR is the interquartile range IQR = Q3 − Q1.

According to the actual measurement data of a handover period, 1458 sampling points of the SS-RSRQ of SCell and 1449 sampling points of the SS-RSRQ of NCell are shown in Figure 7. The SS-RSRQ of the measured data is obtained by a method similar to SS-RSRP. According to the fitting curve, it is found that the measured values of SS-RSRQ gradually decrease and the measured values of NCell gradually increase, that is, the change rule of SS-RSRQ sampling points is consistent with that of SS-RSRP sampling points.

#### 2.3.3. Dynamic Hysteresis Margin of SS-RSRP/SS-RSRQ

**Figure 8:**
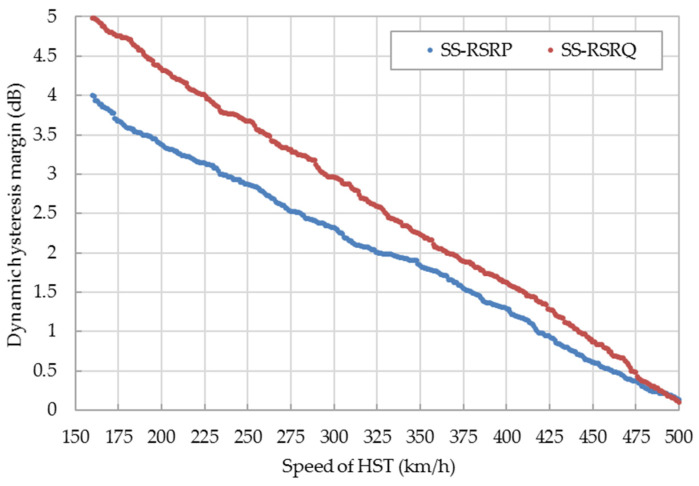
Relationship between the dynamic hysteresis margin of SS-RSRP/SS-RSRQ and the speed of the high-speed train (HST).

In order to suppress ping-pong handover, a hysteresis margin is required between the SCell and the NCell. The default hysteresis margin of 5G is difficult to adapt to 5G-R because the HST is moving at a high speed. The relationships between the dynamic hysteresis margin of SS-RSRP/SS-RSRQ and the speed of HST are shown in Figure 8. The traditional SS-RSRP/SS-RSRQ based on the trigger scheme of the handover generally takes the approximate 5 dB/6 dB as the default hysteresis margin. The hysteresis margin of SS-RSRP/SS-RSRQ is 1.84 dB/2.23 dB when the speed of HST is 350 km/h. With the increase of HST’s speed, the hysteresis margin of SS-RSRP/SS-RSRQ is reduced accordingly to improve the trigger probability of the handover and enhance the success rate of the handover.

**Figure 9:**
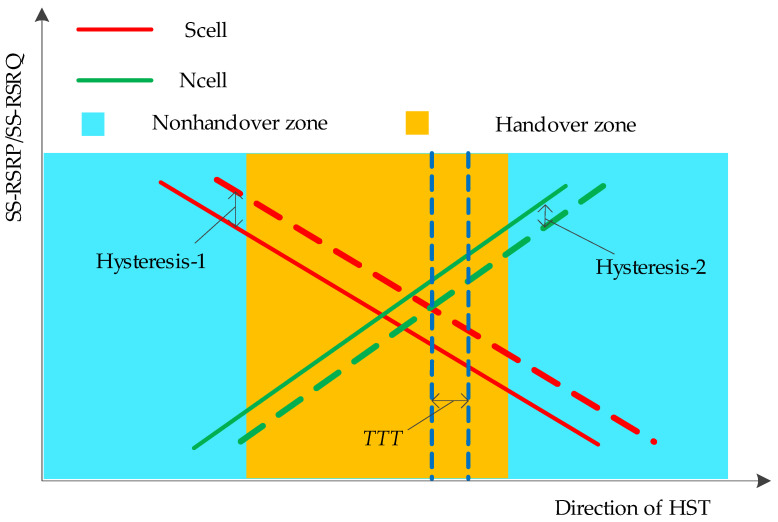
Trigger point of the handover.

Based on the above measurement analysis, the trigger point of the handover is shown in Figure 9. In addition to the measurement results of the Scell and the Ncell, the hysteresis-1, the hysteresis-2, and the time to trigger (*TTT*) are also considered during the process of the handover. In the optimization of wireless communications, these parameters can be adjusted on the station to improve the handover performance, and different values of parameters can adapt to different scenarios.

## 3. Fast Handover Algorithm

### 3.1. Analysis of the Feasibility of the Fast Handover Algorithm

**Figure 10:**
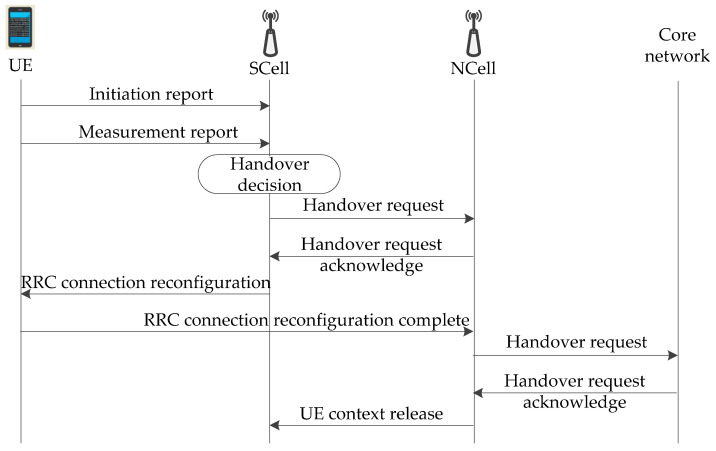
Signaling process of the fast handover algorithm.

The traditional algorithm takes SS-RSRP or SS-RSRQ in the measurement report as the basis of a handover decision. However, the effect of fast fading may cause frequent fluctuation of SS-RSRP or SS-RSRQ in the wireless channel of HSRs, so that UE cannot accurately switch to the appropriate NCell. The chain structure is generally for the network coverage along the rail, and adjacent gNodeBs are alternately distributed on both sides of the rail. When the HST is moving, the order of cells attached by UE is unique. UE’s location information, moving speed, and running direction can be obtained by computing in real time. According to the moving direction of HST, its track is definite. The speed of UE is consistent with the speed of the train. These features are different from the randomness of UE moving range and speed in the scenarios of non-HSRs. In wireless communications, reasonable utilization of these features can solve the problem of the deterioration of performance indexes, such as the success rate of the handover and the delay of the handover in HSRs. The signaling process of the fast handover algorithm in the 5G-R wireless communications for HSRs is shown in Figure 10. The speed and direction of the train, and the location information of UE, are periodically monitored through the initiation report.

### 3.2. Moving Speed of HST

The sampling points of the global positioning system (GPS) and the BeiDou Navigation Satellite System (BDS) are the main data sources for the calculation of the speed of HST. The source of original data needs to undergo the transformation and offset correction of coordinates. Furthermore, abnormal sampling points (above 10 m off the track curve) should be eliminated. The main fields of the calculation of the train’s speed are shown in Table 1. Instantaneous speed $V_{\mathrm{ins}}^{j}$ (*j* = 1, 2, …, *M*) is based on Equation (5). Where, latitude and longitude are (*θ_j_*, *ϕ_j_*), *R* is as the radius of the earth, and Δ*T* is for the interval between two sampling points. The train D2706 runs from Urumqi to Lanzhou.

(5) $$\begin{array}{l} V_{\mathrm{ins}}^{j}=R\times (\arccos(\sin\varphi_{j}\sin\varphi_{\left(j+1\right)}+\cos\varphi_{j}\cos\varphi_{\left(j+1\right)}\cos(\theta_{j}-\theta_{\left(j+1\right)}))\times \\ \;\;\;\;\pi /180{}^{\circ})/(\Delta T/3600) \end{array}$$

In the scenario of HSRs with a speed of 350 km/h and station spacing of 2.5 km, it takes about 26 s for the train to pass through the coverage area of a single station. Based on this, the train’s speed *v* is calculated once within 26 effective cycles of data return, and the equation of calculation is

(6) $$v=\frac{V_{\mathrm{ins}}^{j}+V_{\mathrm{ins}}^{\left(j+1\right)}+\ldots +V_{\mathrm{ins}}^{\left(j+25\right)}}{26}$$

### 3.3. Moving Direction of HST

After the completion of the construction of wireless communications for HSRs, the SCells information attached to UE on the train and the NCells information have strong regularity. The sequences of the handover chain of two UEs in different directions on the same line are opposite. The chain of the handover pointing to Beijing’s direction is defined as positive, and the chain of the handover deviating from Beijing’s direction is reverse. In the handover chain, if gNodeB*_i_* is SCell, gNodeB_(*i*−1)_ and gNodeB_(*i*+1)_ are NCells. At time *t*_1_, the reference signal receiving powers of gNodeB_(*i*−1)_ and gNodeB_(*i*+1)_ are SS-RSRP_(*i*−1)_ and SS-RSRP_(*i*+1)_, respectively. At time *t*_2_, the reference signal receiving powers are SS-RSRP’_(*i*−1)_ and SS-RSRP’_(*i*+1)_, respectively. When Equations (7) and (8) are both satisfied, the train is moving forward and the chain of the handover in the forward is selected. When Equations (7) and (8) cannot be satisfied at the same time, the train moves in reverse and the chain of the handover in reverse is selected. When Equations (7) and (8) satisfy either one, the measurement and calculation are re-performed.

(7) $${\mathrm{SS}-\mathrm{RSRP}}_{\left(i-1\right)}^{\prime }-{\mathrm{SS}-\mathrm{RSRP}}_{\left(i-1\right)}<0$$

(8) $${\mathrm{SS}-\mathrm{RSRP}}_{\left(i+1\right)}^{\prime }-{\mathrm{SS}-\mathrm{RSRP}}_{\left(i+1\right)}>0$$

### 3.4. Process of the Fast Handover Algorithm

The process of the fast handover algorithm includes the speed decision and location recognition of UE, and the weight calculation of the handover.

#### 3.4.1. Speed Decision

When the moving speed *v* of UE is less than *V*_thr_, that is to say that Equation (10) is satisfied. When the service quality of NCell is better than that of SCell, and the duration meets the *TTT*, then the handover of A3 is started. The handover condition of the same frequency band is

(9) $$M_{n}+Ocn-Hys>M_{s}+Ocs+Off$$

where *M*_n_ is the SS-RSRP measurement value of NCell, *M*_s_ is the SS-RSRP measurement value of the SCell, *Ocn* is the offset value of NCell, *Ocs* is the offset value of the source cell, *Hys* is the handover hysteresis, and *Off* is the handover bias. When the moving speed of UE is no less than *V*_thr_, that is, when Equation (11) is satisfied, location recognition is performed.

(10) $$v<V_{\mathrm{thr}}$$

(11) $$v\geq V_{\mathrm{thr}}$$

#### 3.4.2. Location Recognition of UE

In the preliminary study, a UE’s positioning algorithm, which can obtain the real-time location information in the case that the GPS and BDS signals are missing, was proposed based on a fuzzy clustering fingerprint database [30]. Let (*θ_c_*, *ϕ_c_*) be the coordinates of UE’s estimated location, and (*θ_z_*, *ϕ_z_*) be the coordinates of NCell, and the distance between UE and NCell is

(12) $$\begin{array}{l} \Delta D=R\times \arccos(\sin\varphi_{c}\sin\varphi_{z}+\\ \;\;\;\;\cos\varphi_{c}\cos\varphi_{z}\cos(\theta_{c}-\theta_{z}))\times \pi /180{}^{\circ} \end{array}$$

If the distance Δ*D* between the UE and the NCell is not less than *D*_thr_, that is to say, Equation (13) is satisfied, then the location information of UE is continuously updated; if Δ*D* is less than *D*_thr_, that is, Equation (14) is satisfied, then the weight of the handover is calculated.

(13) $$\Delta D\geq D_{\mathrm{thr}}$$

(14) $$\Delta D<D_{\mathrm{thr}}$$

**Figure 11:**
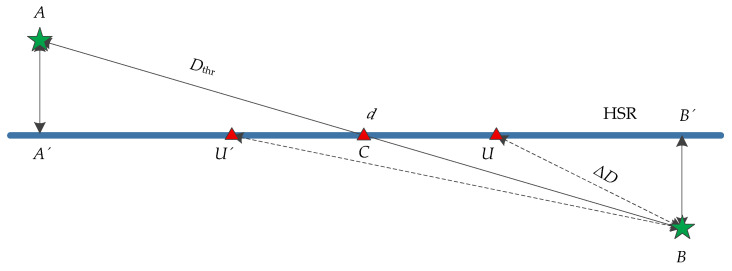
Diagram of the relation between Δ*D* and *D*_thr_.

As shown in Figure 11, suppose the positions of two eNodeBs are *A* and *B*, namely, the distance *d*_1_ = *AB* between eNodeBs, and the distance threshold *D*_thr_ = *d*/2 between UE and the NCell. *AA*’ is the vertical distance between gNodeB and rail, and *AA*’ = *BB*’. *U* and *U*’ are the locations of UE respectively. *BU* and *BU*’ are the distance Δ*D* from UE to NCell. *C* is the intersection point of *AB* and the rail.

#### 3.4.3. Weight Calculation of the Handover

According to a large number of simulation experiments and the statistics measured data, the value range of SS-RSRP acquired by UE in 5G-R wireless communications for HSRs is −156 dBm ~ −31 dBm, and the value of SS-RSRQ ranges from −43 dB to 20 dB. SS-RSRP*_i_* and SS-RSRQ*_i_* are normalized into *P_i_* and *Q_i_*, and SS-RSRP and SS-RSRQ of all sampling points of UE are normalized into sets {*P*} and {*Q*}. The result *d_i_* of the weight calculation of the sampling point *i* is shown in Equation (15), where $W_{i}^{N}$ and $W_{i}^{S}$ are the weights of NCell and Scell, respectively.

(15) $$\begin{array}{l} d_{i}=W_{i}^{N}-W_{i}^{S}=\alpha \times \frac{P_{\left(i+1\right)}-\min(P)}{\max(P)-\min(P)}+(1-\alpha )\times \frac{Q_{\left(i+1\right)}-\min(Q)}{\max(Q)-\min(Q)}-\\ \;\;\alpha \times \frac{P_{i}-\min(P)}{\max(P)-\min(P)}+(1-\alpha )\times \frac{Q_{i}-\min(Q)}{\max(Q)-\min(Q)} \end{array}$$

where *α* (*α* [0,1]) is the weight factor. When *d_i_* > 0, the gNodeB sends the command of the handover; when *d_i_* ≤ 0, the weight of the handover is recalculated.

**Figure 12:**
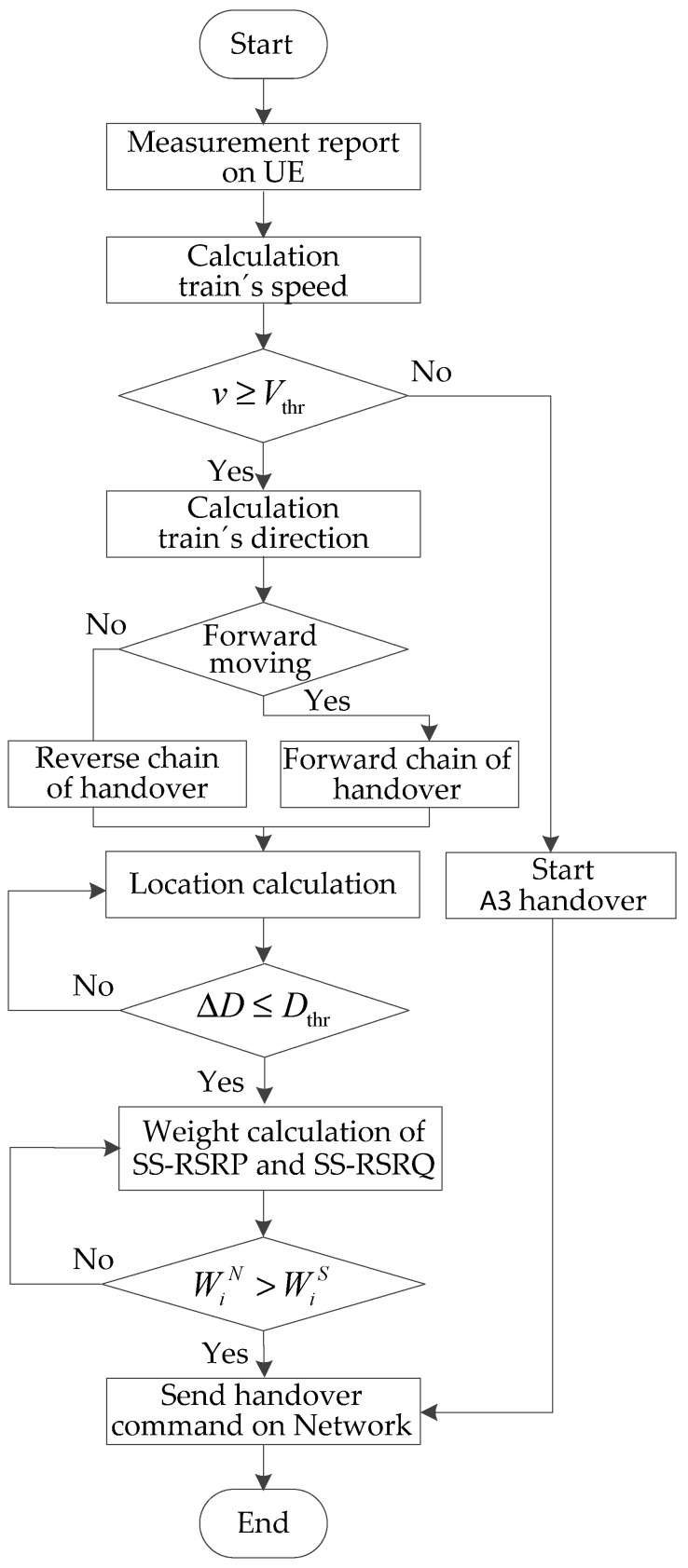
Flow chart of the fast handover algorithm.

Let the delay of the fast handover algorithm be

(16) $$T_{\mathrm{fas}}=\sum_{f=1}^{n}t_{f}$$

where *t_f_* (*f* = 1, 2, …, *n*) is the delay of all interactions of signaling, including the measurement, the judgment, and the execution, which are parts of the end-to-end delay [31]. Compared with the traditional handover algorithm A3, the fast handover algorithm saves the time cost of measurement configuration and periodic reporting. The flow chart of the fast handover algorithm in the 5G-R wireless communications for HSRs is shown in Figure 12.

## 4. Experiment and Analysis

### 4.1. Simulation Parameters

The simulation parameters of the fast handover algorithm for 5G-R refer to the parameter configuration of the comprehensive experimental section of Beijing-Shanghai HSR, as shown in Table 2. Transmission Mode (TM) 3 is the spatial multiplexing of the open loop, which is applied to the wireless environment of UE at high speed. Considering the effect of network coverage, the vertical distance between gNodeBs and rail ranges from 50 m to 200 m in actual deployment. Under the condition of fixed station spacing, if the vertical distance is too close, the grazing angle decreases and the penetration loss increases accordingly. If the vertical distance is too far, the multi-path loss increases, resulting in the effect of poor coverage.

### 4.2. Simulation Results

**Figure 13:**
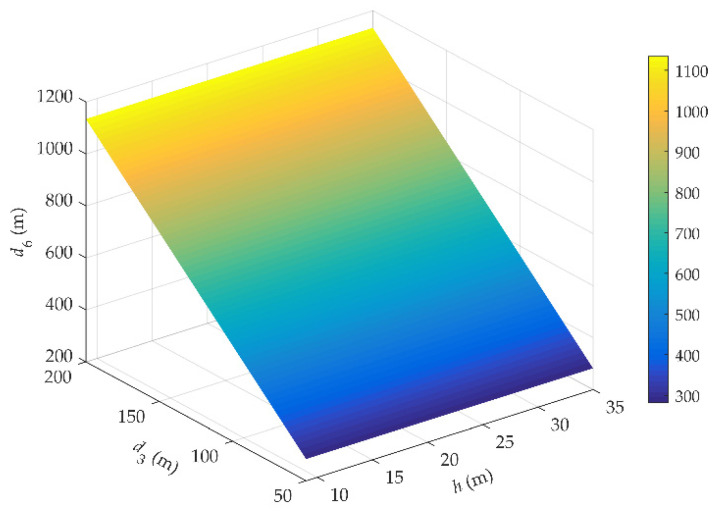
Relationship between *h*, *d*_3_ and *d*_6_.

Figure 13 plots the coverage distance *d*_6_ of gNodeB under different effective height *h* and the horizontal distance *d*_3_. The maximum *d*_6_ on rail of HSRs is not more than 1.2 km. This picture shows that as the *d*_3_ increases, the range of *d*_6_ is extended in the range of 1 km. When the *h* increases, the distance *d*_6_ has hardly improved.

**Figure 14:**
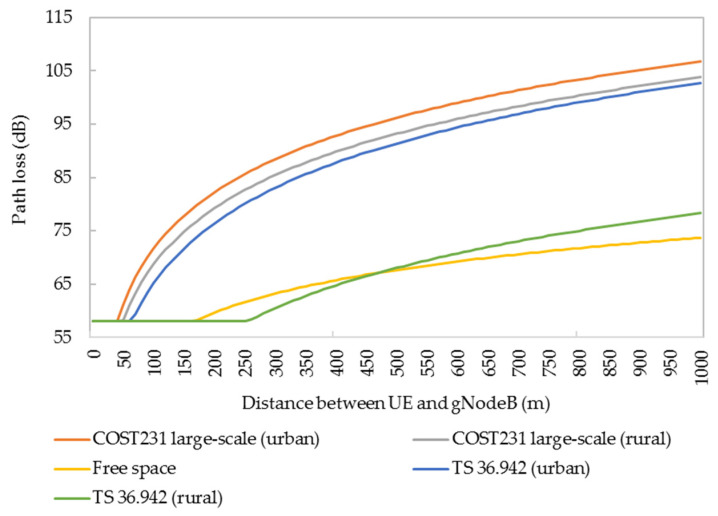
Path losses under different scenarios and different models.

The length of the handover zone is one of the important factors to determine the spacing of gNodeBs. The study of path loss in different scenarios of HSRs is helpful for the rational planning of the handover zone. The power attenuation of wireless signals received under different path-loss models (COST 231 and TS 36.942) and different regional types (suburban and urban areas) are different, as shown in Figure 14. The path loss of suburban terrain and free space in TS 36.942 is relatively low, and the changing trend of path loss in the distance between UE and gNodeB is basically the same in the other four cases.

**Figure 15:**
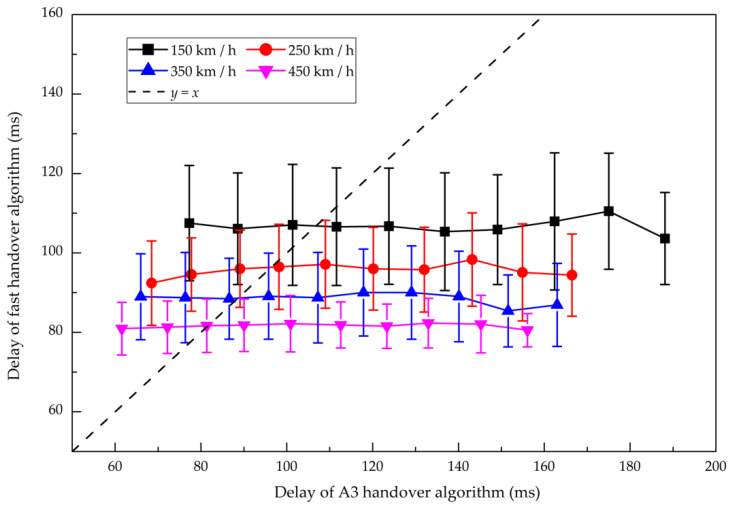
Comparison of the delays of the handover between the fast handover algorithm and the A3 handover algorithm. Marks represent the bin averages of different handover delay intervals, and bars correspond to the standard deviation in each bin.

Figure 15 displays the delays of the handover with two algorithms at different speeds of HST. Since the process of the fast handover algorithm is optimized, the delay of the control plane is greatly shortened. This is basically consistent with the results of the theoretical analysis. When the speed increases gradually, the delay of the fast handover algorithm is steadily improved due to the optimized measurement mechanism and execution process. Compared with the A3 handover algorithm at speeds 150 km/h, 250 km/h, 350 km/h and 450 km/h, the delays of the fast handover algorithm are reduced by 1.39%, 1.94%, 2.26%, and 2.54%, respectively. The advantage of the fast handover algorithm makes full use of the characteristics of HSRs, such as known train running line and recognizable handover chain, thus reducing the redundant overhead of time.

**Figure 16:**
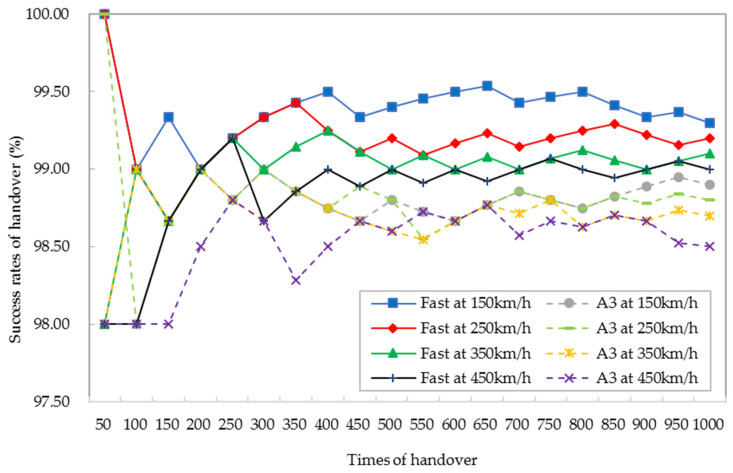
Comparison of the success rates of the handover between the fast handover algorithm and the A3 handover algorithm.

The success rate of the handover, which directly affects the key indexes such as drop rate and throughput, is one of the important indexes to measure wireless communications. The expression of the success rate of the handover is

(17) $$P_{\mathrm{han}}=1-\frac{N_{\mathrm{fai}}}{N_{\mathrm{att}}}$$

where *N*_fai_ is the number of failed handovers, and *N*_att_ is the number of attempted handovers. When the times of the handover exceed 450, the success rates of the handover of both algorithms tend to be stable as shown in Figure 16. Taking 1000 times of the handover as an example, the success rate of the handover is increased by 0.42% on average at the four speeds of HST. Based on the above analysis, the inter-macro cell of handover and the conventional scheme of handover reach the same success probability of handover [17]. The proposed fast handover algorithm has a more stable and superior success rate of the handover. The fast handover algorithm and inter-macro-cell handover [32] reach the same effect: they not only reduce the delay of the handover but also improve the success rate of the handover.

## 5. Conclusions

In the scenario of HSRs, the effective coverage distance of gNodeB has little relation with the height of base station, but it is strongly correlated with the horizontal distance between gNodeB and rail. This is fundamentally different from the non-HSRs. The power attenuation values of wireless signals are unlike under the diverse path loss models and various region types, so it is necessary to distinguish and apply them reasonably in the construction of 5G-R. When both large-scale fading and shadow fading are taken into account, the design of overlapping coverage areas between gNodeBs along the rail is relatively complex.

The process of the handover in 5G-R can be optimized through the planning of handover zone, the estimate of the speed of HST and the location recognition of UE, and the weight calculation of the handover. It can effectively reduce the delay of the handover and improve the success rate of the handover. With the increase of speed of a train in the range of 150 km to 450 km, the delay of the fast handover algorithm is significantly reduced. The speed of HST is inversely proportional to the success rate of handover, and the proposed algorithm can improve the success rate of handover. In the future, with continuously increasing train running speeds, fast handover algorithms will become more important. As 6G technology is being developed, the fast algorithm can be further applied for HSRs with distinct characteristics such as a definite trajectory of movement, a knowable range of running speed, and an identifiable handover chain.

## Figures and Tables

**Table 1:** Running speed of HST.

Train Number	Data Return Time	Longitude	Latitude	Instantaneous Speed (km/h)
D2706	9 December 2020 17:06:41	97.286705	40.104435	209.46
	9 December 2020 17:06:42	97.287155	40.103947	239.06
	9 December 2020 17:06:43	97.287613	40.103508	224.84
	9 December 2020 17:06:44	97.288086	40.103127	210.33
	9 December 2020 17:06:45	97.288544	40.102764	201.95
	9 December 2020 17:06:46	97.288933	40.102238	241.93
	9 December 2020 17:06:47	97.289375	40.101746	238.98
	9 December 2020 17:06:48	97.289810	40.101322	215.76
	9 December 2020 17:06:48	97.290268	40.100941	207.20

**Table 2:** Configuration of parameters.

Parameters	Value
Frequency	2.6 GHz
Bandwidth	100 MHz
*n*Tx/*n*Rx	4/4
TM	3
*d*_1_	1.5 km
*h*	25 m
Antenna mechanical dip angle	3°
Antenna electronic dip angle	6°
Antenna gain	21.5 dB
Minimum coupling loss	58 dB
*V*_thr_	120 km/h
*D*_thr_	0.9 km
*α*	0.75
Moving speed of HST	150/250/350/450 km/h

## Data Availability

Data available on request from the authors.
